# LGA infants display early catch down growth in length and weight without epigenetic changes

**DOI:** 10.1186/1687-9856-2015-S1-P106

**Published:** 2015-04-28

**Authors:** Valentina Chiavaroli, Wayne Cutfield, José Derraik, Pan Zengxiang, Sherry Ngo, Allan Sheppard, Susan Craigie, Fredrik Ahlsson

**Affiliations:** 1 Liggins Institute, University of Auckland, Auckland, New Zealand; 2 Department of Women's and Children's Health, Uppsala University, Uppsala, Sweden

## Aims

To evaluate the growth patterns of infants born large-for-gestational-age (LGA) from birth to age 1 year compared to those born appropriate-for-gestational-age (AGA). In addition, we aimed to investigate possible epigenetic changes associated with being born LGA.

## Methods

Seventy-one singleton infants born at term were classified by birth weight as AGA (10th-90th percentile; n=42) and as LGA (>90th percentile; n=29). Post-natal follow-up until age 1 year was performed with clinical assessments at 3, 6, and 12 months. Assessments included measurement of infants’ weight, length, ponderal index, BMI, as well as head, chest, and abdominal circumference. A subgroup of 38 infants (17 AGA and 21 LGA) was selected for genome-wide DNA methylation analysis. Umbilical cord was collected at birth, and methylation profile on umbilical tissue was analysed using the Illumina Infinium 450K methylation array.

## Results

At birth, the LGA group had greater birth weight (P<0.0001), length (P<0.0001), head circumference (P<0.0001), ponderal index (P=0.02), BMI (P<0.0001), chest and abdominal circumferences (P=0.04 and P=0.007, respectively) than AGA newborns. At the age of 3 months, LGA infants still presented greater weight (P<0.0001), length (P=0.006), BMI (P=0.02), as well as head (P=0.004) and abdominal (P=0.04) circumferences than AGA peers. However, by age 6 months there were no more anthropometric differences between the two groups. This was due to higher length increment in AGA than LGA infants between 0-6 months (18.3 vs 15.1 cm; P<0.0001), whereas length increment was identical in both groups between 6-12 months (7.7 vs 7.7 cm; P=0.96). For the genome-wide analysis, more than 485,000 DNA methylation sites covering 99% of human NCBI Reference Sequence (RefSeq) genes were examined at birth, but no differences were found between LGA and AGA infants.

## Conclusion

Despite being born oversized at birth, LGA infants displayed early catch-down growth (i.e. slower length velocity), so that by the age of 6 months LGA infants were of similar length and BMI as AGA infants. In addition, no epigenetic differences in genome-wide methylation were found in those born LGA.

